# Parkinson's Disease and Cryptogenic Epilepsy

**DOI:** 10.1155/2016/3745631

**Published:** 2016-09-05

**Authors:** Andre Y. Son, Milton C. Biagioni, Dorian Kaminski, Alec Gurevich, Britt Stone, Alessandro Di Rocco

**Affiliations:** ^1^The Marlene and Paolo Fresco Institute for Parkinson's & Movement Disorders, NYU Langone Medical Center, New York University School of Medicine, New York, NY, USA; ^2^Department of Neurology, Baylor Scott & White Healthcare, Austin, TX, USA

## Abstract

Epilepsy is an uncommon comorbidity of Parkinson's disease (PD) and has been considered not directly associated with PD. We present five patients (3 men and 2 women; ages 49–85) who had concomitant PD and cryptogenic epilepsy. Although rare, epilepsy can coexist with PD and their coexistence may influence the progression of PD. While this may be a chance association, an evolving understanding of the neurophysiological basis of either disease may suggest a mechanistic association.

## 1. Introduction

Parkinson's disease (PD) is traditionally considered primarily a subcortical disorder with late cortical involvement. There is, however, mounting evidence of considerable cortical involvement, even in the early stages of PD [1, 2]. The damage seems to involve, at least in part, the simultaneous processes of both the diffuse progressive deposition of alpha-synuclein [1] and the secondary biological changes that disrupt neural connectivity [3]. These processes reinforce the concept of dysfunctional neural networks as a potential basis of symptomatology in PD [4].

Considering PD in this regard allows one to posit the potential associations it may have with other diseases involving dysfunctional neural circuits. Epilepsy, for example, is such a disease but to date has not been considered associated with PD [5]. We report five subjects with PD followed up at our institution with comorbid cryptogenic epilepsy.

## 2. Methods

An internal PD registry was queried and individual movement disorders physicians were interviewed to determine cases of coexisting PD and epilepsy. Among the data of over 500 PD patients screened, seven were initially identified with concomitant epilepsy and their medical records were carefully reviewed. All seven patients were followed up for PD at our institution. None had personal or family histories of neurological disorders, including, but not limited to, PD and epilepsy. None had a history of deep brain stimulation, intracranial hemorrhage, cerebral vascular accident, or other brain injuries, and all patients underwent extensive workup that included MRI imaging. This retrospective chart review was considered exempt for consent by the local institutional review board. Two patients were not included in the report: one with atypical features that challenged the diagnosis of idiopathic PD and the other with a poorly documented seizure history.

## 3. Cases

Of the five patients reported below, three (Cases 1–3) had epilepsy before developing PD while Cases 4 and 5 developed seizures after being diagnosed with PD. Relevant patient characteristics are summarized in Table 1.

### 3.1. Patient 1

This is a 66-year-old right-handed man who at age 30 experienced his first generalized tonic-clonic (GTC) seizure. He was maintained on phenytoin with a seizure frequency of about 1 GTC seizure every 7 years until age 53 when he was switched to levetiracetam after developing osteoporosis. At age 57, he developed right-hand tremor, bilateral leg heaviness, and slurred speech followed by masked facies, micrographia, and later left-hand tremor and bilateral leg tremor and was eventually diagnosed with PD. He was started on carbidopa-levodopa three times daily, with good clinical response and control of his motor symptoms. At age 63, levetiracetam was switched to lamotrigine to address fatigue. However, soon after the switch, he experienced several GTC and complex partial events. Despite multiple antiepileptic drug (AED) adjustments, for the next 4 years, his seizure frequency was 1–3 per month, characterized by GTC and complex partial events. At age 64, coinciding with a period with frequent seizures and changes in AEDs, his PD symptoms began to worsen despite an increase in PD medications. He started having more frequent freezing episodes, falls, worsened postural stability, cognitive decline, hallucinations, and decreased independence with activities of daily living (ADLs). At last observation at age 66, 9 years after his PD diagnosis, he was unable to care for self, had moderately severe dementia, and moved to an assisted living facility as he could no longer care for himself.

### 3.2. Patient 2

This is a 49-year-old right-handed man who at age 43 was diagnosed with cryptogenic epilepsy. His epilepsy was characterized by complex partial events with several instances of secondary generalization and it was well controlled on carbamazepine and clonazepam. At age 48, he presented with left-hand rest and postural tremors, loss of dexterity, and hyposmia and was diagnosed with PD. He has been followed up for the past 2 years, but, at last observation at age 49, he complained of tremor in social settings and tip-of-tongue phenomenon. Otherwise, he is independent with his ADLs and does not complain of cognitive deficits. His seizures continue to remain well controlled and he has not been started on any anti-Parkinson's medications.

### 3.3. Patient 3

This is a 74-year-old right-handed man who experienced his first seizure, a GTC event, in his 20s. He was well controlled for years with phenobarbital and phenytoin and later with carbamazepine. At age 72 he became aware of “slowing down” and a year later on neurological evaluation he was found to have bilateral resting and action tremors, stooped posture, masked facies, and axial bradykinesia and was diagnosed with PD and started on carbidopa-levodopa with good clinical response. At last observation at age 74, he was participating in physical therapy and had improvement in tremor, bradykinesia, back pain, and anxiety on carbidopa-levodopa.

### 3.4. Patient 4

This is a 79-year-old left-handed woman who at age 64 started dragging her right leg and developed right-hand tremor that led to a diagnosis of PD, treated with carbidopa-levodopa with robust clinical response. At age 70, she started to experience episodes of altered mental status (AMS) that lasted a few hours and frequent nocturnal events, with screaming and body jerks. These episodes were initially interpreted as cognitive episodes, or due to orthostatic hypotension or vascular insults, until the workup excluded the presence of clinically relevant autonomic dysfunction or vascular injuries. At age 73, after an episode of AMS preceded by visual and olfactory phenomena, she was diagnosed with epilepsy, with a video-EEG demonstrating bilateral temporal spikes and cortical irritability. She was then started on lamotrigine with resolution of the recurrent nocturnal events. After another AMS episode, lamotrigine was increased to 75 mg and levetiracetam 250 mg was added, with marked improvement and no recurrence of AMS. While her seizures were controlled, her PD progressed rapidly and she developed severe motor fluctuations and cognitive and psychiatric symptoms. By age 76, she had moderately severe balance impairment, falls, episodes of dystonia, paranoia, hallucinations, REM behavioral disorder, and cognitive fluctuations and required substantial assistance with ADLs. At last observation at age 79, she was entirely dependent for ADLs requiring two-person assist to walk or rise from sitting and had moderate dementia, more frequent periods of confusion and disorientation, and frequent hallucinations, and her fluctuations became more severe and resistant to medication adjustment, with frequent off periods and troublesome dyskinesia.

### 3.5. Patient 5

This is an 86-year-old right-handed woman who at age 76 developed hand tremors (left  >  right) and loss of dexterity and was diagnosed with PD. She presented to our institution at age 81 at a point when she had bilateral hand tremors (left  >  right) and lip tremor. Her motor symptoms remained well controlled with no cognitive or psychiatric problems, until age 82, when she started to experience frequent paroxysmal events of AMS lasting anywhere from minutes to several hours with no associated focal neurological findings that were initially interpreted as PD associated cognitive episodes. After a video-EEG demonstrated left temporal slowing and sharp slow wave complexes in association with one of the AMS episodes, she was diagnosed with epilepsy and was started on levetiracetam with significant improvement, with three more episodes of AMSs, over the following three years. At last observation at age 86, she had been seizure-free for at least a year while her PD had slowly progressed but she remained independent with her ADLs and without cognitive or other nonmotor complications.

## 4. Discussion

Although being limited in number, ours is one of the largest reported case series of Parkinson's disease and epilepsy. Among the five subjects reported, two developed seizures after they had been diagnosed with PD, while the other three had a recognized seizure disorder prior to developing PD symptoms. All patients were thoroughly investigated for secondary forms of epilepsy with no structural lesions on MRI, concomitant diseases, or drugs or toxin exposure that could cause seizures.

There was no specific clinical profile that emerged from the review of these cases, but patients with poor seizure control had a more aggressive progression of their PD and had cognitive complications. This may be a reflection of summative effects of seizures in an already affected neurological background, although the additional burden of epilepsy medication in patients already exposed for years to neurological drugs may also play a role. The presence of PD may also have an effect on the severity of seizures, especially in patients whose PD manifested after the onset of seizures. It is also important to remark the challenges in establishing a new diagnosis of seizures in an established PD patient, as illustrated by Cases 4 and 5, in which the seizure events were initially interpreted as complications of PD.

Our study also seems to confirm the lack of a direct relationship between PD and epilepsy [5]. While we identified 7 cases in our cohort of over 500 PD subjects with seizure disorders, the overall prevalence is similar to the prevalence of epilepsy observed in the general elderly population greater than age 65, which is estimated to be between 10 and 11 individuals per 1,000 [6], suggesting that our observed rate of comorbid epilepsy in PD patients may occur by chance.

Our series has several limitations, including its retrospective nature, reliance on chart entries by variable practitioners, and the heterogeneous population. Despite these limitations, our series raises awareness to a number of challenges associated with the coexistence of PD and cryptogenic epilepsy.

While the few cases detected in this retrospective review support a chance association between PD and epilepsy, the study was inspired by the growing evidence of early cortical involvement in PD. Not only can Lewy bodies be detected in different cortical areas, often in earlier stages of the disease [1], but there are other early cortical changes in PD, including regional structural changes with cortical thinning and architectural changes [1, 7] and changes in network connectivity [4]. While these changes do not seem to herald a higher incidence of epilepsy in PD, it is possible that they may be in part responsible for more complex clinical manifestations of epilepsy in PD.

In conclusion, while PD and epilepsy can cooccur simply by chance, we believe that this case series should raise awareness that their coexistence may pose unique clinical challenges, including difficulty in diagnosing or correctly interpreting the nature of paroxysmal neurological episodes and a possible modifying effect that PD and epilepsy may have on each other.

## Figures and Tables

**Table 1:** Clinical and demographic characteristics.

	1	2	3	4	5
Age (years)	66	49	74	79	86
Sex	Male	Male	Male	Female	Female
Order of Dx	Epilepsy then PD	Epilepsy then PD	Epilepsy then PD	PD then epilepsy	PD then epilepsy
Epilepsy duration (years)	36	6	50	6	4
Type of seizures	Complex partial, GTC	Complex partial, secondarily generalized	Complex partial, GTC	Complex partial	Complex partial
PD duration (years)	10	1	1	15	10
UPDRS-III at PD Dx	9.5	10	21	28.5	26
H&Y stage at PD Dx	1	1	2.5	2.5	2.5
UPDRS-III at epilepsy Dx	N/A	N/A	N/A	27	21
H&Y stage at epilepsy Dx	N/A	N/A	N/A	2	2
UPDRS-III at last visit	51	23	20	51	36
H&Y stage at last visit	3	2	2.5	4	2
Most recent antiepileptic medication(s)	Eslicarbazepine	Carbamazepine and clonazepam	Carbamazepine	Levetiracetam and lamotrigine	Levetiracetam
Most recent LEDD (mg)	750	N/A	390	796	780

Dx, diagnosis; GTC, generalized tonic-clonic; PD, Parkinson's disease; UPDRS, Unified Parkinson's Disease Rating Scale; H&Y, Hoehn and Yahr; N/A, not applicable; LEDD, levodopa equivalent daily dose.
